# Delivering Safe Surgical Care While Simultaneously Caring for Patients With COVID-19; Assessment of Patient Selection, Volume and Outcomes in a Tertiary Care Hospital

**DOI:** 10.3389/ijph.2023.1605640

**Published:** 2023-03-27

**Authors:** Anja Domenghino, Roxane Diane Staiger, Fariba Abbassi, Miquel Serra-Burriel, Kim Leutwyler, Guillaume Aeby, Matthias Turina, Christian Alexander Gutschow, Pierre-Alain Clavien, Milo Alan Puhan

**Affiliations:** ^1^ Department of Surgery and Transplantation, University Hospital Zürich, Zurich, Switzerland; ^2^ Epidemiology, Biostatistics and Prevention Institute (EBPI), University of Zurich, Zürich, Switzerland; ^3^ University of Zurich, Zürich, Switzerland

**Keywords:** pandemic, quality assessment, complications, surgical quality, Clavien-Dindo classification, Comprehensive Complication Index (CCI^®^)

## Abstract

**Objectives:** Compare patient selection and postoperative outcomes after surgical treatment for gastrointestinal disorders before and during the SARS-CoV-2 pandemic.

**Methods:** We assessed gastrointestinal surgeries conducted at a tertiary center from 2017–2021 for differences in patient populations and procedures before (up to February 2020) and during the pandemic (March 2020 to December 2021). We analyzed mortality, Intensive Care Unit (ICU) length of stay, admission to ICU and postoperative complications for complex procedures using descriptive statistics and regression models.

**Results:** 7309 procedures were analyzed, showing a caseload reduction in March and October 2020, but no statistical evidence for fewer overall procedures overall. Population characteristics differed with lower Body Mass Indices in 2020 and 2021, more patients smoking and with diabetes treated in 2020. There was no increased mortality, ICU length of stay and in 1,144 complex procedures assessed low overall morbidity at 90 days postoperative.

**Conclusion:** Delivering surgical care while treating patients for COVID-19 in the same hospital was safe. Healthcare officials should consider continuing surgical care during future health crises as consequences of limiting surgical treatment for gastrointestinal disorders may be fatal for patients.

## Introduction

The SARS-CoV2 pandemic has disrupted surgical services worldwide. The millions of procedures cancelled are likely to have far reaching, not yet captured consequences for patients, healthcare providers and systems alike (1). The measures taken vary greatly between countries, which offers the opportunity to analyze the different strategies of dealing with the pandemic in hospitals. This may help to guide us on how to handle public health crises in the future.

In the United Kingdom (UK) and Wales over 2.5 million procedures were cancelled in 2020 and 2021 as resources were reallocated to accommodate hospital beds and intensive care unit (ICU) space for SARS-CoV-2 positive patients (2). At the beginning of the pandemic, it was feared intubation or laparoscopic surgery could lead to an increased spread of the aerosol born virus and increase the infection risk of healthcare providers. Public Health England and some surgical societies therefore decided on drastic limitations of aerosol generating procedures (3). The pandemic also gave room to more drastic measures such as treating acute appendicitis conservatively or under spinal anesthesia (4), awake major abdominal surgery in Italy (5), treating acute cholecystitis and other biliary disorders conservatively in the UK, which lead to worse postoperative outcomes (6, 7). Due to the reduction in theatre and endoscopy capacity procedures of cancer patients were often delayed. This caused later detection of bowel cancer or change from curative to palliative treatment intent (8, 9). Researchers predicting an increase in the number of deaths due to colorectal and esophageal cancer about 16% and 6% up to 5 years after diagnosis (10).

The UK developed so called COVID-19 clean sites where only SARS-CoV-2 negative patients were operated on. This led to good results with no higher complication rate and no infection with SARS-CoV-2 during the hospital stay and its ensuing complications. However, when patients tested positive for SARS-CoV-2 on admission, the operation was postponed; those consequences where not accounted for (11). There is no doubt that avoiding a perioperative infection with SARS-CoV-2 is of the upmost importance as this increases mortality and postoperative complications (12). However, the delay of life saving procedures has far reaching consequences, not all of which can be measured yet, if ever. Further, there were reports on improvements of postoperative outcomes, especially concerning infectious aspects, attributed to better hygiene protocols (13, 14).

Switzerland is exemplary for a country where pandemic restrictions were relatively mild and medical needs of the population were deemed at least as important as the combat of the pandemic. The only ban for non-urgent medical procedures took place during the first lockdown between 16 March 2020, and 26 April 2020, as imposed by the Federal Council (15). Furthermore, there was some necessity to delay or cancel surgeries due to lacking ICU capacity afterwards, which led to a reduction in all in-hospital stays of 5.8% (16). However, not many of the procedures were performed later and no catch-up effect was observed (16, 17). Additionally, there were less patients seeking medical treatment in Switzerland due to fear of getting infected with SARS-CoV-2 or to relieve stress on the healthcare system (18). Most cantons of Switzerland did not establish COVID-19 clean sites and hospitals continued to treat patients in need for emergency surgery, cancer resections or transplantation while also treating SARS-CoV-2 positive patients.

The aim of this study was to investigate how the pandemic affected the selection of surgical patients, procedures performed and postoperative outcomes of patients undergoing emergency or elective gastrointestinal surgery in the third largest tertiary center in Switzerland.

### Research in Context

#### Evidence Before this Study

We searched PubMed and EMBASE for peer-reviewed studies evaluating the effect of the pandemic and associated lockdown measures on surgical outcomes in gastrointestinal surgery on 7 April 2022. Search terms were related to “COVID-19” (“SARS-CoV-2,” “coronavirus disease 2019,” “COVID-19”), (“gastrointestinal surgery,” “gastrointestinal” AND “surgery”) and “outcome.” We used no language or time restrictions. We identified additional records through manual searches of references and screened over 1,346 studies using the Rayyan web application, which can be accessed through https://rayyan.qcri.org for screening, deleting 148 duplicates. Two hundred and forty-five studies compared surgical outcomes in pre-pandemic settings with a timespan during the pandemic. Through full text screening, we identified 116 that included patients receiving emergency or elective surgical treatment for gastrointestinal disorders and compared a pre pandemic cohort with one during the pandemic. Most studies compared either March 2020 or March to June 2020 with either the same period of the year before or the same calendar period before the pandemic. However, none of the studies included all data for the course of consecutive years including one and a half years of pandemic data. Many studies presented only short-term outcomes and complications. Evidence from countries using separated COVID-19 free pathways and postponing or cancelling gastrointestinal procedures already showed that this can cause severe harm to patients.

#### Added Value of this Study

This is the first study to compare postoperative outcomes after emergency and elective surgical treatment for gastrointestinal disorders between a pre-pandemic and a pandemic cohort, which includes data from a timespan of five years. We, therefore, factor in variability between years and, within years, between months due to circumstances other than the pandemic. We also present complication data for 90 days postoperative for selected complex procedures to ensure that more long-term effects are captured. These data were collected in a tertiary center that continued to treat COVID-19 patients during the pandemic, in a country where restrictions where relatively mild and medical needs of the population were deemed at least as important as the combat of the pandemic. The study showed that also under these circumstances surgical care can be safe.

#### Implications of all the Available Evidence

Surgery for emergency and elective gastrointestinal disorders, especially cancer and solid organ transplantation, is an essential part of public health care. Thus, it should be weighted accordingly in health officials’ strategic evaluation of reducing hospital services due to extraordinary circumstances. Our data highlights the feasibility of safe surgical care for gastrointestinal disorders during a pandemic which may help rebuilding patients’ trust in the health care system for a future Public Health Emergency of International Concern.

## Methods

### Patient Population and Data Collection

Data from a tertiary center in Zurich, Switzerland were included from 1 January 2017, to 31 December 2021. Included were patients that underwent any emergency or elective surgical procedure for gastrointestinal disorders (Supplementary Table S1 lists the most common procedures). Patients who did not receive an intervention or patients who actively rejected the general consent form were excluded. There were no further exclusion criteria for the overall analysis to give a complete picture of all patients treated during this time. In a clarification of responsibility from the cantonal ethical commission of Zurich (BASEC Nr. Req-2021-00159) approval was not required according to Swiss law.

We retrospectively obtained patient and outcome data that was collected prospectively from the hospital information system and the administrative database. The procedures were sorted using the codes of the official Swiss classification system for surgical procedures for the elected years (CHOP). For complex procedures bearing a higher risk for postoperative complications one of the investigators (either AD, FA, GA, or KL) checked the outcomes and recalculated the Comprehensive Complication Index (CCI®) by retrospectively reading through the individual patient records. This included liver, pancreatic, gastric, and esophageal resections, as well as resections of the rectum.

We investigated specific time points during the pandemic that affected the course in Switzerland according to the times the government released national measures or ordinances: the first lockdown starting on 16 March 2020 (19), the reduction of gatherings to a maximum of 15 people and beginning of mask obligatory on 19 October 2020 (20), and the beginning of using the national certificate, indicating whether one was vaccinated or recovered from SARS-CoV-2 on 13 September 2021 (21). Details on the hospital processes, measures taken to protect patients and employees against infections with SARS-CoV-2, and guidelines on handling SARS-CoV-2 patients at our university hospital are described in the Supplementary Material (Supplementary Material—Policies, Provisions and SARS-CoV-2 patients at the Tertiary University Hospital).

### Outcomes

Our primary outcomes were overall mortality, length of ICU stay, probability of being admitted to the ICU, and postoperative morbidity of those receiving the most complex procedures. Secondary outcomes included postoperative wound infections and pneumonia. Patient characteristics were age, sex, and the Patient Clinical Complexity Level (PCCL), a measure of the cumulative effect of a patient’s comorbidities that is calculated for each episode of care and important for reimbursement in the Diagnosis-Related Groups system of Switzerland (22). We used the established International Classification of Diseases (ICD-10) codes for Switzerland to screen patients for diabetes, chronic kidney disease, chronic lung disease, cardiovascular disease, and COVID-19 infection. To assess overall morbidity the CCI® was used (23, 24). This internationally used and validated overall morbidity measure is based on the Clavien-Dindo classification (CDC) (25, 26). The index captures one or multiple complications in one patient. This mirrors the effect of the overall complication burden on a patient reflected on a scale between 0 (no complication) and 100 (death). It allows morbidity assessments over time as complication after discharge can easily be accumulated to the previous CCI®. This enabled the comparison of long-term outcomes.

### Statistical Analysis

We used descriptive statistics to analyze participants’ baseline characteristics and the above-mentioned outcomes. Continuous variables are presented as median with minimum and maximum, categorical, or ordinal variables as frequencies (N) and percentages (percentage). Missing values are reported where applicable. We report *p*-values for differences in baseline characteristics for the respective years and used multivariable quasipoisson regression to assess the impact of the individual years on the PCCL.

Multivariable linear regression was used for the outcomes of ICU length of stay, wound infections, and pneumonia, and multivariable logistic regression was used for the outcomes of mortality and probability of ICU admission to assess potential influence of the individual years. . All models were adjusted for age, sex, and PCCL as an indicator of overall health status. Results from the regression analysis are reported with their corresponding *p*-values. For the complex procedures, we analyzed the overall CCI® 90 days postoperatively and used linear multivariable regression to account for any differences between the years, including age, sex, PCCL, comorbidities, BMI, and smoking status.

We analyzed for period effects of the most impactful times of the pandemic. March to May 2020 (this is during the first wave in Switzerland and after the first lockdown), November 2020 (when mask obligatory was introduced in any public space) and September 2021 (when the national certificate was introduced). The distribution of baseline characteristics (age, sex, PCCL, comorbidities, and BMI) in the population during the individual months and for the specific procedures conducted during these months were assessed to account for differences in numbers during the years.

We performed several sensitivity analyses to ensure robustness of study findings. First, for the assessment of ICU admission probability, we excluded all patients that received a gastric bypass or elective hernia operations, as those are procedures with low possibility of ICU stay and significant differences in numbers during the pandemic years. Second, we excluded all deaths to eliminate the high weight of the CCI® of 100 from the overall analysis. R version 4.0.4 (2021-02-15) was used for all analyses (27).

## Results

### Study Population and Procedures

There was a total of 8,341 procedures performed that required an in-hospital stay. Of those 1,032 patients (12.4%) rejected the general consent form to make their data available for research (Table 1). Differences in the baseline characteristics were found over the years such as lower mean Body Mass Indices (BMI) in the years 2020 and 2021 and more patients reported smoking in 2020. When adjusted for age and sex in the multivariable regression model no evidence for differences in the PCCL for the individual years were found. There was statistical evidence of more patients with diabetes treated in 2020 (*p* = 0.004) which remained significant in a multivariable regression adjusted for age and sex (*p* < 0.01). While there was an apparent reduction in elective surgical cases after the lockdown and the mask obligatory during the first two big waves in Switzerland (Supplementary Figure S1), there was no statistical evidence showing fewer overall procedures in the years 2020 and 2021. Compared to the previous 3 years a lower number of gastric bypass surgeries and elective hernia procedures but more emergency cholecystectomies and liver and pancreatic resections were performed in 2020 and 2021 (Supplementary Table S1). Assessing the individual surgeries in March 2020 and November 2020 overall fewer procedures were done. However, this did not affect the number of appendectomies or cholecystectomies (Supplementary Tables S2, S3; Supplementary Figures S2–S5).

**TABLE 1:** Baseline characteristics of patients operated on at the tertiary center between 2017–2021. Switzerland.

	2017	2018	2019	2020	2021	*p*-value
Patients treated	1,632	1,728	1,764	1,573	1,644	
Patients consented	N = 1,475	N = 1,495	N = 1,542	N = 1,362	N = 1,435	
Age mean (SD)	52.8 (17.0)	53.5 (17.5)	53.0 (17.1)	53.7 (16.8)	53.4 (17.2)	0.620
Sex						0.231
M	790 (53.6%)	822 (55.0%)	852 (55.3%)	728 (53.5%)	822 (57.3%)	
W	685 (46.4%)	673 (45.0%)	690 (44.7%)	634 (46.5%)	613 (42.7%)	
PCCL						0.085
0	718 (48.7%)	727 (48.6%)	731 (47.4%)	640 (47.0%)	646 (45.0%)	
1	13 (0.88%)	15 (1.00%)	17 (1.10%)	16 (1.17%)	23 (1.60%)	
2	118 (8.00%)	124 (8.29%)	156 (10.1%)	137 (10.1%)	150 (10.5%)	
3	262 (17.8%)	261 (17.5%)	261 (16.9%)	226 (16.6%)	292 (20.3%)	
4	364 (24.7%)	368 (24.6%)	377 (24.4%)	343 (25.2%)	324 (22.6%)	
Comorbidities						
Diabetes						0.004
No	1,318 (89.4%)	1,320 (88.3%)	1,340 (86.9%)	1,161 (85.2%)	1,279 (89.1%)	
Yes	157 (10.6%)	175 (11.7%)	202 (13.1%)	201 (14.8%)	156 (10.9%)	
Kidney						0.694
No	1,310 (88.8%)	1,309 (87.6%)	1,361 (88.3%)	1,189 (87.3%)	1,270 (88.5%)	
Yes	165 (11.2%)	186 (12.4%)	181 (11.7%)	173 (12.7%)	165 (11.5%)	
Lung						0.056
No	1,376 (93.3%)	1,390 (93.0%)	1,402 (90.9%)	1,255 (92.1%)	1,340 (93.4%)	
Yes	99 (6.71%)	105 (7.02%)	140 (9.08%)	107 (7.86%)	95 (6.62%)	
Heart						0.966
No	993 (67.3%)	1,002 (67.0%)	1,033 (67.0%)	924 (67.8%)	955 (66.6%)	
Yes	482 (32.7%)	493 (33.0%)	509 (33.0%)	438 (32.2%)	480 (33.4%)	
COVID-19	0	0	0	11 (0.81%)	8 (0.56%)	
BMI mean (SD)	27.5 (7.43)	27.2 (7.27)	27.3 (7.08)	26.7 (6.38)	26.3 (6.98)	<0.001
Smoking						<0.001
No	1,157 (78.4%)	989 (66.2%)	1,142 (74.1%)	980 (72.0%)	1,090 (76.0%)	
Yes	225 (15.3%)	243 (16.3%)	247 (16.0%)	327 (24.0%)	249 (17.4%)	
*Missing*	93 (6.31%)	263 (17.6%)	153 (9.92%)	55 (4.04%)	96 (6.69%)	
Immuno-suppression						<0.001
Yes	160 (10.8%)	105 (7.02%)	154 (9.99%)	152 (11.2%)	139 (9.69%)	
No	1,156 (78.4%)	971 (64.9%)	1,105 (71.7%)	1,007 (73.9%)	1,111 (77.4%)	
*Missing*	159 (10.8%)	419 (28.0%)	283 (18.4%)	203 (14.9%)	185 (12.9%)	

There were 8,341 procedures conducted between 1 January 2017, and 31 December 2021, 7,309 of which baseline characteristics of treated patients are presented.

**FIGURE 1:**
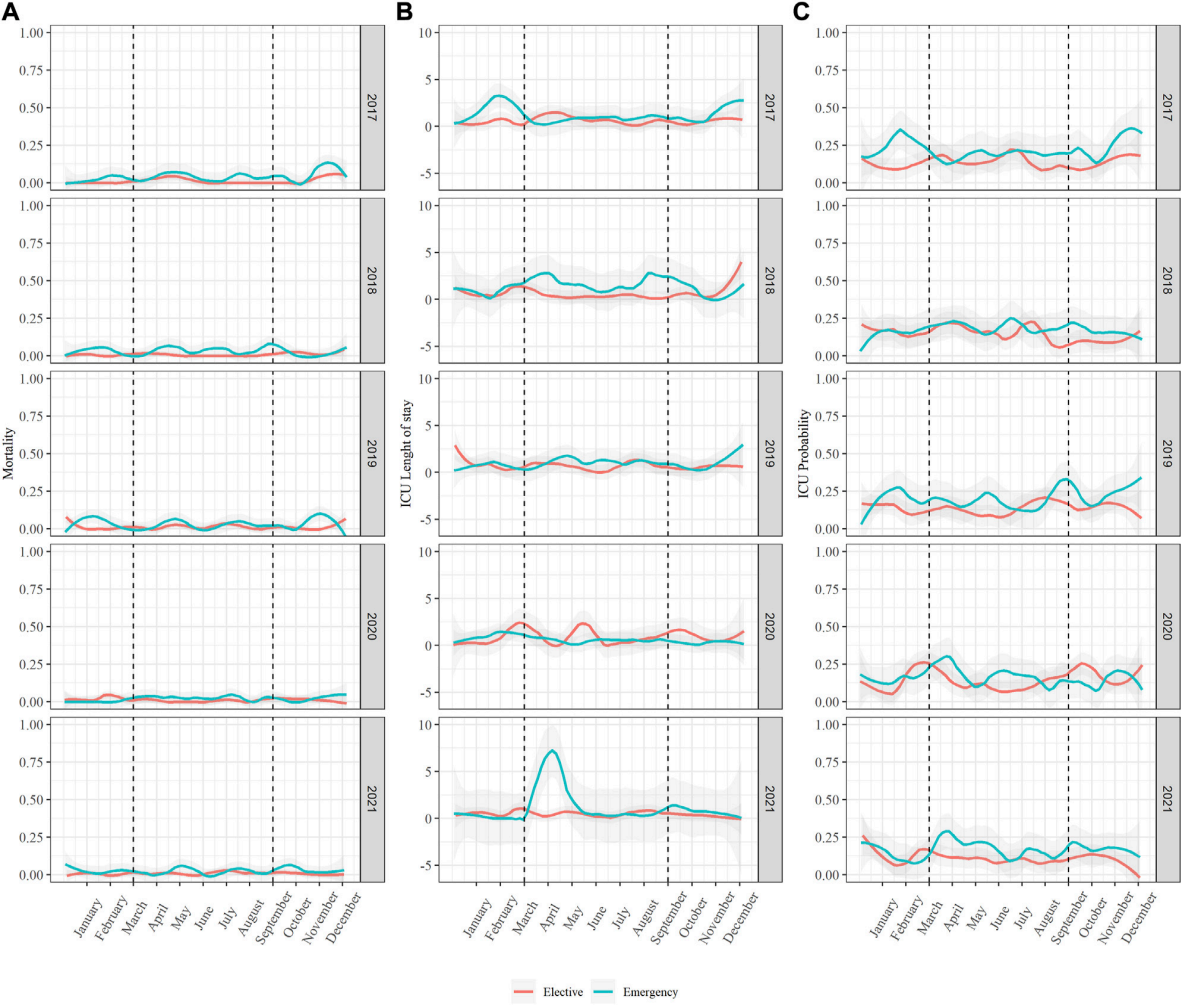
Mortality, ICU length of stay and ICU probability in the entire study population. Switzerland, 2017–2021. Data of all 7,309 performed procedures was analyzed between 1 January 2017, and 31 December 2021, looking at **(A)** Mortality, **(B)** ICU length of stay and **(C)** ICU probability.

There was no statistical evidence for differences in mortality or ICU length of stay over the years when adjusted for sex, age, and PCCL. We saw an increase in ICU length of stay in 2021 between March and May that we did not see in any of the four previous years (Figure 1). When analyzing the characteristics of the patient population during this time and comparing it to the population in March to May of the previous 4 years there was no statistical evidence for differences except the already mentioned lower BMI in 2020 and 2021 (*p* < 0.005). There was also no statistical evidence for a difference in procedures and their complexity during this time except for the reduction in gastric bypass surgeries (*p* = 0.023).

There was low evidence for a lower ICU probability in 2021 (*p* < 0.5) when adjusting for sex, age, and PCCL in the multivariable regression with a corresponding drop in ICU probability at the end of this year. To ensure this did not indicate a deferral of patient on the Intermediate Care station (IMC) we analyzed IMC probability as well, there was no indication of a corresponding increase during this time. In sensitivity analysis, excluding all patients who received an elective hernia or gastric bypass procedure the ICU probability in 2021 remained lower (*p* < 0.01) when compared to previous years.

### Complex Procedures—Patient Characteristics and Postoperative Complications

**FIGURE 2:**
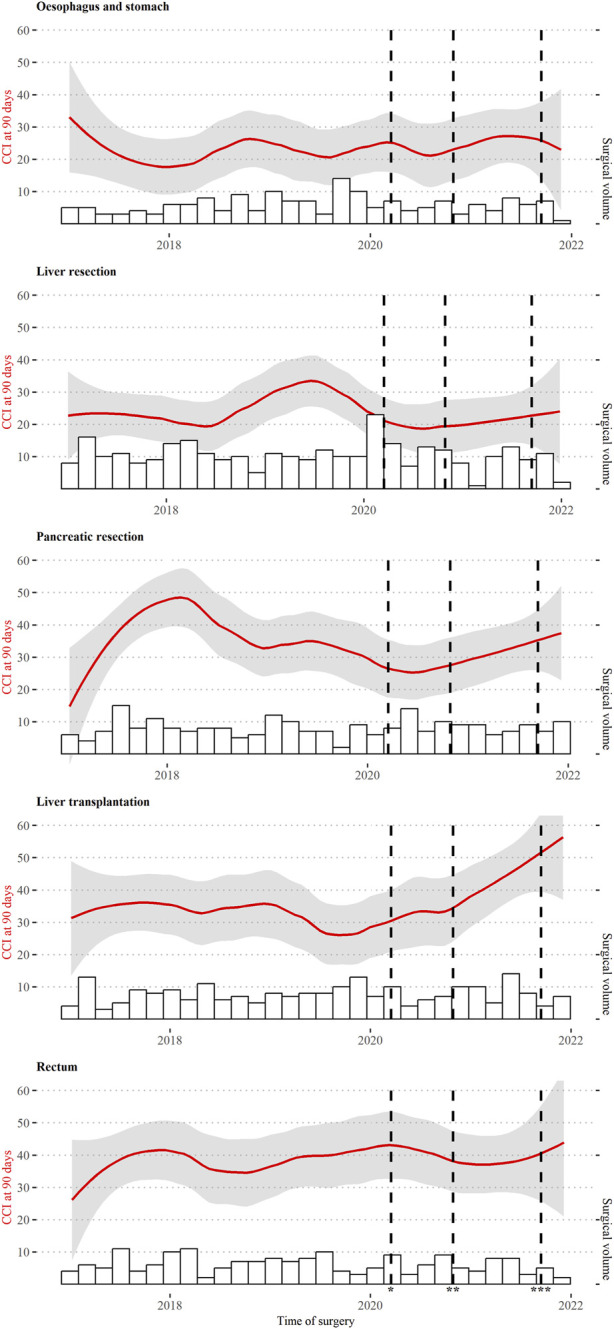
CCI® at 90 days after complex procedures. Switzerland, 2017–2021. All 1,144 performed complex procedures between 1 January 2017, and 31 December 2021, including esophagus and stomach, liver resection, pancreatic resection, liver transplantation and rectum resection. * 16 March 2020, first lockdown, ** 19 October 2020, mask obligatory, *** 19 September 2021, implementation of national certificate.

For the more complex procedures like esophagus and stomach resections, liver resections, pancreatic resections, liver transplantations and rectum resections, a total of 1,144 procedures, the postoperative complications at 90 days were analyzed (Figure 2). There was some variation over the course of the 5 years, both in the number of procedures as well as overall morbidity (CCI®). There was no reduction of performed complex procedures during the pandemic years. Statistical evidence for an increase in 90 days CCI® after liver transplantation in 2021 (*p* = 0.019) was found. There were twice as many deaths after liver transplantation in that year (seven compared to three or four in previous years), although no statistically significant difference in mortality when conducting a regression adjusting for age and sex. When looking at the patient characteristics of those receiving a liver transplant there were some differences including fewer patients suffering from diabetes, but more of male patients and patients who reported smoking in 2021 and fewer patients with a high PCCL in 2019 (Supplementary Table S4). There was also evidence for an increase in the CCI® after pancreatic resection in 2018, but it needs to be noted that more complex patients (PCCL 4 at 75%) were treated during that time. The variations in liver, esophageal and gastric resections did not show changes over the years.

### Postoperative Complications—Wound Infections and Pneumonia in the Total Population

**FIGURE 3:**
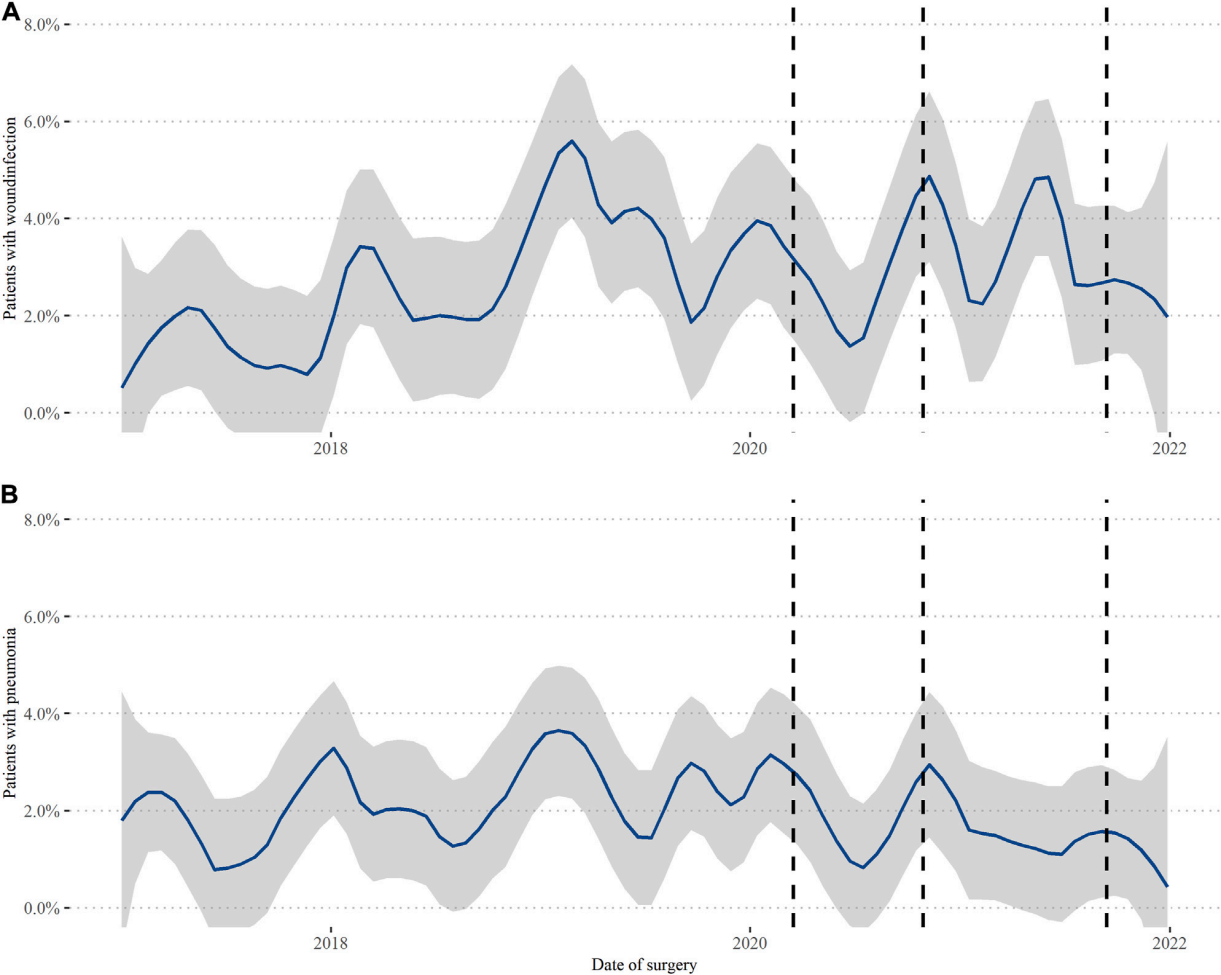
Wound infections and pneumonia in the total population. Switzerland, 2017–2021. Data of all 7,309 performed procedures was analyzed between 1 January 2017, and 31 December 2021, looking at **(A)** postoperative wound infections and **(B)** pneumonia. The lines again represent 16 March 2020, first lockdown, 19 October 2020, mask obligatory, and 19 September 2021, implementation of national certificate.

When assessing the proportion of all 7,309 patients who suffered from a wound infection over the years there seem to be visible drops around the times when Switzerland issued a new national decree as described above (Figure 3). In the linear regression adjusted for age, sex, and PCCL there was statistical evidence of more wound infections in 2019–2021 when compared to the 2 years before (2017: 20/1475 (1.4%), 2018: 38/1495 (2.5%), 2019: 61/1542 (4.0%), 2020: 40/1362 (2.9%), 2021: 46/1435 (3.2%), 2019: *p* = 0.001, 2020 *p* = 0.01, 2021 *p* = 0.001). There was no statistical evidence for significant changes over the total years in pneumonia cases, even if there was some decrease in 2021 (2017: 29/1475 (2.0%), 2018: 31/1495 (2.1%), 2019: 40/1542 (2.6%), 2020: 29/1362 (2.1%), 2021: 19/1435 (1.3%)).

## Discussion

We analyzed a total of 5 years of surgical outcome data to detect possible impact by nationwide SARS-CoV-2 measures on postoperative outcome. We observed a reduction in case load during the most impactful times of the pandemic, but overall, the number of procedures performed per year did not decline, and postoperative outcomes were not worse during the pandemic compared to three pre-pandemic years. Delivering healthcare for gastrointestinal diseases can be feasible and safe even when simultaneously caring for SARS-CoV-2 infected patients in the same hospital.

It was inevitable for surgical departments worldwide to cancel and postpone elective surgeries to save resources for SARS-CoV-2 infected patients. However, patients suffering from gastrointestinal diseases, also face consequences of not getting treated in a timely manner (9, 10, 28). We did not see a reduction in emergency or complex, elective procedures during the pandemic years in this tertiary center. The obligatory responsibility by law to accept every case referred from other institutions or emergency care services of Swiss centers were not lifted during the pandemic. Numbers of emergency cholecystectomies, elective liver and pancreatic resections were even increasing during the months of high COVID-19 case numbers. Other European institutions reported lower numbers of performed emergency surgeries with worse outcomes during the pandemic. Hospitals from Spain reported a 59% decrease of emergency surgery from middle of March to end of April 2020 (29). Researchers in Torino (Piedmont, Italy), one of the most affected areas in Italy, found postponing surgery in colorectal cancer patients was not justified (30). In the UK several studies reported that treating acute cholecystitis and other biliary disorders conservatively led to more complicated surgeries and worse outcomes (6, 7), they concluded that treatment of these patients should not be hold off. Compared to the UK, Italy and Spain, Switzerland had quite mild measures for containing the pandemic which could explain why we did not see such a reduction in emergency cases. They could also indicate a reduction of possibilities to receive the urgent and necessary treatment for cholecystitis and pancreatic or liver cancer at other centers in Switzerland. We also did not see a reduction in appendectomies, something reported by many other authors, also in Switzerland (31, 32). There was no change in patients baseline characteristics of those who did receive surgery, as reported by other countries (33). The one surgical field where we saw a significant reduction in case numbers for the years 2020 and 2021 was in bariatrics, which also explains the reduction in BMI we noticed in the baseline characteristics. These interventions were almost completely shut down during the first and second wave of the pandemic. This is not surprising as these are elective procedures for patients at highest risk of adverse outcomes of SARS-CoV-2 infections. In the UK, nearly all (97.8%) elective bariatric surgeries were postponed in April 2020, after the first peak of the COVID-19 pandemic (34).

We found continuously good outcomes after abdominal surgeries, even while, at times, treating more patients than pre pandemic and adhering to standard, evidence-based procedures for gastrointestinal disorders. Our study demonstrated that while there is some fluctuation in mortality, ICU length of stay and ICU probability over the analyzed 5 years, there was no negative impact that could be directly attributed to the pandemic. We only saw that ICU probability seemed to be lower than ever before at the end of 2021 in both groups elective and emergency abdominal surgeries. There did not seem to be a difference in baseline characteristics or composition of procedures that would explain this reduction and we could credit this to doctors being more resistant in submitting patients to the ICU when not utterly necessary, as ICU beds were rare during this time. This could be rated as beneficial as ICU stays can have severe impacts on the mental state of patients and their families and are very costly (35). Even in the most complex interventions, there was no significant change in the CCI®, which is a very sensitive tool to determine overall morbidity. In Spain, non-inferior surgical outcomes during the COVID pandemic indicated that resuming elective surgery is safe even in high-risk patients (36). However, there were data, also from Spain, analyzing surgical outcomes in over 5,000 patients that saw a likelihood of greater failure-to-rescue than pre-pandemic controls (37).

Appendicitis is the abdominal disease and surgical procedure where most data are available to assess the impact of the pandemic. In the UK, the pandemic has resulted in significant modifications to the pre-operative work-up and surgical approach to patients undergoing emergency appendectomy. Whilst the proportion of patients with complicated appendicitis has increased post-lockdown, overall clinical outcomes remained similar (38). In Switzerland, morbidity and reoperation rate did not change (31, 32). We saw that continuing surgery for gastrointestinal diseases can be safe and feasible, as long as strategies recommended by surgical societies to minimize the risk of perioperative SARS-CoV-2 transmission are followed. Data from the Netherlands also described that the performance of emergency general and oncological surgical procedures remained stable in a cohort of 1,399 patients (39). While data form an international multicenter study recommended to provide COVID-19–free surgical pathways (complete segregation of the operating theater, critical care, and inpatient ward areas) (40), there was also a study from within the NHS that only found a minimal impact of the pandemic on postoperative morbidity and mortality (41). Furthermore, COVID-19-free surgical pathways are not feasible for many centers.

Some positive implications were found that could guide us in providing safe care in extraordinary times. For one, we did see some reduction in surgical site infections and even hospital-acquired pneumonia around the times of governmental decrees. Even though those results were not statistically significant, they still highlight the importance of attention and follow-through of guidelines of healthcare personal. There were results from cardiac surgery in the UK that showed a significant reduction in postoperative sternal wounds during the pandemic, so this effect is not to be neglected (14).

During the pandemic not only treatment but also diagnosis of surgical gastrointestinal disorders, especially, but not exclusively, cancerous diseases were significantly impacted. Total colorectal cancer diagnoses and screening diagnoses were both significantly lower in the pandemic interval in Denmark (42) and the UK, with a reduction in the number of people referred, diagnosed, and treated for colorectal cancer (43). Colleagues from the UK showed that premature cancer deaths resulting from diagnostic delays during the first wave of the COVID-19 pandemic in the UK would result in significant economic losses. On a per-capita basis, this impact is, in fact, greater than that of deaths directly attributable to COVID-19 (44). A 3-month delay to surgery across all stage 1–3 cancers is estimated to cause >4,700 attributable deaths per year in England (45). Which concludes that support both resource allocation and the prioritization of time-critical health services directly affected in a pandemic, such as cancer care.

Another aspect we might be able to address with our data is the fear of patients to seek help at a hospital, afraid of being infected with SARS-CoV-2, which could also lead to worse health outcomes as seen in other countries (46). In Switzerland we did see good long-term outcomes after emergency and complex cancer surgeries. This could encourage surgeons and doctors to feel safe when performing procedures and help patients and the general public to feel comfortable to come to the hospital and go to outpatient clinics for elective care and preventive screening.

This study has several limitations. We had to exclude patients who actively rejected the general consent form. This might have introduced some selection bias, however the proportion of patients consenting did not differ across periods, so it is unlikely to affect the comparisons of interest. Due to the high number of patients included, the outcome measure CCI® at 90 days postoperatively could not be checked and recalculated for all patients; we had to concentrate on complex cases, more prone to have complicated courses. However, as we chose five procedures representing the general elective surgeries most prominent operations and analyzing their CCI® for a total of over 1,144 patients we believe that they represent a sample of the overall population most at risk for postoperative complications and consequences from delayed treatment. Furthermore, we limited the observed time to 90 days postoperatively again due to feasibility. A 90-day period for some procedures is not enough to detect all complications and even longer-term outcomes would be of interest and might be a focus of further research. This study focuses on gastrointestinal and general surgeries conducted in one department of a single tertiary care center. Thus, there is some limitation to the generalizability of our data. But the university hospital did not have policies substantially different from those of other larger hospitals, and we did not restrict the broad patient population treated at this university hospital so it can be expected that the results apply more broadly to larger hospitals offering general and abdominal surgery.

### Conclusion

The selection and outcomes of patients undergoing surgery under pandemic conditions did not differ from pre-pandemic years. In our tertiary care center, we did not find a decrease in number or postoperative outcome in emergency appendectomies or cholecystectomies, as reported in many facilities in other countries. Delivering surgical care while treating patients for COVID-19 in the same hospital appears to be feasible and safe. Hospital and healthcare officials can include this data when they must consider whether to continue surgical care during future public health crises.

## Abbreviations

CCI®, Comprehensive Complication Index; CDC, Clavien-Dindo Classification; CHOP, Swiss classification system for surgical procedures; HPB, Hepato-pancreatic-biliary; ICU, Intensive Care Unit; PCCL, Patient Clinical Complexity Level; UK, United Kingdom.
