# Supplementary material for: Delivering Safe Surgical Care While Simultaneously Caring for Patients With COVID-19; Assessment of Patient Selection, Volume and Outcomes in a Tertiary Care Hospital
Source: Int J Public Health. 2023 Mar 27;68:1605640. doi: 10.3389/ijph.2023.1605640 (PMC10083247; doi:10.3389/ijph.2023.1605640)
Supplement: Supplementary file 1 [file DataSheet1.PDF]

Delivering safe surgical care while simultaneously caring for Patients with COVID-19;

Assessment of Patient Selection, Volume and Outcomes in a Tertiary Care Hospital

Supplementary Files

Supplementary Files - Index

Supplementary Methods 3
Policies, provisions, and SARS-CoV-2 patients at the tertiary care center 3
Supplementary Figures and Tables 4
Table S1 4
Table S2 4
Table S3 5
Table S4 5
Table S5 5
Table S6 6
Table S7 6
Table S8 7
Table S9 7
Figure S1 8
Figure S2 9
Figure S3 10
Figure S4 11
Figure S5 12

Supplementary Methods

Policies, provisions, and SARS-CoV-2 patients at the tertiary care center

We treated over 1805 SARS-CoV-2 positive patients until the 31.12.2021 and 195 died with or because of the disease. Of the 8400 employees, 1165 tested positive up to the end of our study period. We held “Townhall Meetings” to inform employees about the Coronavirus as early as February 04, 2020. The hospital personnel received over 65 newsletters between February 2020 and December 2021, beginning with the information on how to disinfect hands and stay at home orders whenever you developed symptoms. All the time the stringent adherence on hygiene policies was promoted, including wearing masks whenever possible. On February 28, 2020, all events for groups and conferences were banned. Visitors were prohibited on March 12, 2020, and when allowed again from May 30, 2020, on, only limited to a certain number and strictly controlled. There was a mask obligatory beginning on March 20, 2020, and there were mask and disinfection stations at all access points. From March 21, 2020, until April 27, 2020, all interventions were limited to urgent cases, which did include surgeries for cancer and all diseases that could possibly reduce life expectancy or cause permanent disability. Patients were tested for SARS-CoV-2 through a Polymerase-Chain-Reaction (PCR) Test when they were admitted beginning from March 31, 2020. There were dedicated wards, operation theaters and ICU wards for SARS-CoV-2 patients always. Vaccination was offered to employees and high-risk patients beginning January 05, 2021, and from May 18, 2021, on all unvaccinated personnel was tested at least once a week. Whenever the patient tested positive, the elective procedure was postponed. Twelve patients receiving elective procedures and seven receiving emergency ones were operated on, even though they tested positive at admission.

Supplementary Figures and Tables

Table S1

Table S1: Procedures conducted between 2017 and 2021, Switzerland						
	2017	2018	2019	2020	2021	P Value
Procedures	N=1475	N=1495	N=1542	N=1362	N=1435	
Emergencies						
Appendectomy	153 (10.4%)	151 (10.1%)	167 (10.8%)	143 (10.5%)	137 (9.55%)	0.830
Cholecystectomy	39 (2.64%)	70 (4.68%)	58 (3.76%)	63 (4.63%)	72 (5.02%)	0.009
Anorectal	41 (2.78%)	52 (3.48%)	50 (3.24%)	37 (2.72%)	65 (4.53%)	0.051
Bowel	65 (4.41%)	73 (4.88%)	67 (4.35%)	59 (4.33%)	60 (4.18%)	0.911
Other	197 (13.4%)	184 (12.3%)	194 (12.6%)	168 (12.3%)	168 (11.7%)	0.754
Elective						
Colorectal	217 (14.7%)	219 (14.6%)	236 (15.3%)	196 (14.4%)	202 (14.1%)	0.912
Liver and pancreatic resections	125 (8.47%)	113 (7.56%)	112 (7.26%)	139 (10.2%)	119 (8.29%)	0.044
Liver Transplantation	45 (3.05%)	42 (2.81%)	55 (3.57%)	43 (3.16%)	48 (3.34%)	0.805
Kidney, incl. pancreatic transplantation	96 (6.51%)	108 (7.22%)	88 (5.71%)	104 (7.64%)	106 (7.39%)	0.222
Gastroesophageal	65 (4.41%)	79 (5.28%)	101 (6.55%)	76 (5.58%)	84 (5.85%)	0.131
Gastric bypass	128 (8.68%)	112 (7.49%)	111 (7.20%)	82 (6.02%)	87 (6.06%)	0.031
Hernias	245 (16.6%)	232 (15.5%)	230 (14.9%)	158 (11.6%)	187 (13.0%)	0.001
Other*	212 (14.4%)	227 (15.2%)	244 (15.8%)	249 (18.3%)	262 (18.3%)	0.009

* other procedures include but are not limited to endocrine resections like thyroidectomy or resection of the adrenal gland, splenectomies, Hyperthermic intraperitoneal chemotherapy (HIPEC) procedures, tracheostomies, vascular procedures, removal of lesions around the abdominal wall and, laparoscopic biopsies

Table S2

Table S2 – Procedures conducted in March each year between 2017 and 2021, Switzerland						
	2017	2018	2019	2020	2021	P Value
Procedures	N=158	N=132	N=119	N=108	N=152	
Emergencies						
Appendectomy	12 (7.59%)	20 (15.2%)	14 (11.8%)	17 (15.7%)	19 (12.5%)	0.242
Cholecystectomy	8 (5.06%)	4 (3.03%)	1 (0.84%)	3 (2.78%)	5 (3.29%)	0.400
Anorectal	3 (1.90%)	2 (1.52%)	6 (5.04%)	4 (3.70%)	6 (3.95%)	0.421
Bowel	6 (3.80%)	9 (6.82%)	1 (0.84%)	3 (2.78%)	3 (1.97%)	0.098
Other	19 (12.0%)	18 (13.6%)	12 (10.1%)	8 (7.41%)	14 (9.21%)	0.537
Elective						
Colorectal	27 (17.1%)	18 (13.6%)	22 (18.5%)	19 (17.6%)	24 (15.8%)	0.858
Liver and pancreatic resections	10 (6.33%)	13 (9.85%)	9 (7.56%)	14 (13.0%)	7 (4.61%)	0.121
Liver Transplantation	5 (3.16%)	3 (2.27%)	3 (2.52%)	3 (2.78%)	2 (1.32%)	0.866
Kidney, incl. pancreatic transplantation	5 (3.16%)	6 (4.55%)	3 (2.52%)	5 (4.63%)	12 (7.89%)	0.228
Gastroesophageal	6 (3.80%)	9 (6.82%)	10 (8.40%)	5 (4.63%)	7 (4.61%)	0.459
Gastric bypass	19 (12.0%)	12 (9.09%)	5 (4.20%)	2 (1.85%)	7 (4.61%)	0.006
Hernias	30 (19.0%)	23 (17.4%)	21 (17.6%)	13 (12.0%)	21 (13.8%)	0.516
Other*	15 (9.49%)	15 (11.4%)	22 (18.5%)	21 (19.4%)	32 (21.1%)	0.021

* other procedures include but are not limited to endocrine resections like thyroidectomy or resection of the adrenal gland, splenectomies, Hyperthermic intraperitoneal chemotherapy (HIPEC) procedures, tracheostomies, vascular procedures, removal of lesions around the abdominal wall and, laparoscopic biopsies

Table S3

Table S3 – Procedures conducted in November each year between 2017 and 2021, Switzerland						
	**2017**	**2018**	**2019**	**2020**	**2021**	**P Value**
**Procedures**	**N=125**	**N=118**	**N=135**	**N=99**	**N=128**	
**Emergencies**						
**Appendectomy**	13 (10.4%)	9 (7.63%)	15 (11.1%)	11 (11.1%)	13 (10.2%)	0.899
**Cholecystectomy**	5 (4.00%)	6 (5.08%)	2 (1.48%)	5 (5.05%)	8 (6.25%)	0.318
**Anorectal**	3 (2.40%)	6 (5.08%)	3 (2.22%)	4 (4.04%)	7 (5.47%)	0.539
**Bowel**	8 (6.40%)	3 (2.54%)	7 (5.19%)	3 (3.03%)	5 (3.91%)	0.604
**Other**	9 (7.20%)	13 (11.0%)	23 (17.0%)	12 (12.1%)	10 (7.81%)	0.082
**Elective**						
**Colorectal**	21 (16.8%)	20 (16.9%)	21 (15.6%)	14 (14.1%)	15 (11.7%)	0.766
**Liver and pancreatic resections**	10 (8.00%)	5 (4.24%)	8 (5.93%)	13 (13.1%)	13 (10.2%)	0.120
**Liver Transplantation**	2 (1.60%)	3 (2.54%)	9 (6.67%)	3 (3.03%)	6 (4.69%)	0.262
**Kidney, incl. pancreatic transplantation**	12 (9.60%)	7 (5.93%)	12 (8.89%)	9 (9.09%)	7 (5.47%)	0.633
**Gastroesophageal**	7 (5.60%)	4 (3.39%)	8 (5.93%)	10 (10.1%)	8 (6.25%)	0.362
**Gastric bypass**	9 (7.20%)	10 (8.47%)	8 (5.93%)	5 (5.05%)	7 (5.47%)	0.826
**Hernias**	22 (17.6%)	20 (16.9%)	19 (14.1%)	9 (9.09%)	19 (14.8%)	0.428
**Other**	14 (11.2%)	21 (17.8%)	23 (17.0%)	18 (18.2%)	23 (18.0%)	0.535

Table S4

Logistic regression model for mortality over the years. Switzerland, 2017 - 2021			
**Predictors**	**Odds Ratios**	**CI**	**p-value**
(Intercept)	0.00	0.00 – 0.00	<0.001
2018	0.75	0.42 – 1.31	0.312
2019	0.90	0.52 – 1.54	0.688
2020	0.67	0.36 – 1.19	0.177
2021	0.81	0.46 – 1.43	0.478
Age	1.02	1.01 – 1.03	0.003
Sex (female)	1.17	0.80 – 1.68	0.416
PCCL	11.71	6.36 – 25.51	<0.001
Observations	7309		
R2	0.053		

Table S5

Linear regression model for ICU length of stay over the years. Switzerland, 2017 - 2021			
**Predictors**	**Odds Ratios**	**CI**	**p-value**
(Intercept)	-0.49	-0.93 – -0.05	0.029
2018	0.13	-0.21 – 0.47	0.462
2019	0.13	-0.22 – 0.47	0.468
2020	0.07	-0.28 – 0.42	0.696
2021	-0.02	0.46 – 1.43	0.929
Age	-0.00	-0.01 – 0.00	0.171
Sex (female)	-0.32	-0.54 – -0.10	0.004
PCCL	0.60	0.53 – 0.67	<0.001
Observations	7309		
R2/ R2 adjusted	0.045 / 0.044		

Table S6

Logistic regression model for ICU probability over the years. Switzerland, 2017 - 2021			
**Predictors**	**Odds Ratios**	**CI**	**p-value**
(Intercept)	0.00	0.00 – 0.00	<0.001
2018	0.95	0.75 – 1.19	0.644
2019	1.01	0.81 – 1.27	0.908
2020	0.87	0.69 – 1.10	0.238
2021	0.77	0.61 – 0.97	0.027
Age	1.01	1.00 – 1.01	0.005
Sex (female)	0.82	0.70 – 0.95	0.009
PCCL	2.97	2.75 – 3.22	<0.001
Observations	7309		
R2	0.252		

Table S7

Table S4 – Patient characteristics of patients receiving a liver transplant at the USZ. Switzerland, 2017 - 2021						
	**2017**	**2018**	**2019**	**2020**	**2021**	**P Value**
**Patients**	N=42	N=44	N=53	N=44	N=49	
**Age, mean (SD)**	55.7 (9.82)	54.0 (11.9)	54.2 (12.8)	53.5 (9.46)	53.7 (13.1)	0.911
**Sex**						0.575
**M**	27 (64.3%)	30 (68.2%)	38 (71.7%)	28 (63.6%)	38 (77.6%)	
**W**	15 (35.7%)	14 (31.8%)	15 (28.3%)	16 (36.4%)	11 (22.4%)	
**PCCL**						0.227
**2**	0 (0.00%)	0 (0.00%)	1 (1.89%)	1 (2.27%)	0 (0.00%)	
**3**	0 (0.00%)	2 (4.55%)	5 (9.43%)	1 (2.27%)	1 (2.04%)	
**4**	42 (100%)	42 (95.5%)	47 (88.7%)	42 (95.5%)	48 (98.0%)	
**Comorbidities**						
**Diabetes**						0.001
**no**	24 (57.1%)	16 (36.4%)	15 (28.3%)	16 (36.4%)	32 (65.3%)	
**yes**	18 (42.9%)	28 (63.6%)	38 (71.7%)	28 (63.6%)	17 (34.7%)	
**Kidney**						0.795
**no**	30 (71.4%)	31 (70.5%)	41 (77.4%)	29 (65.9%)	36 (73.5%)	
**yes**	12 (28.6%)	13 (29.5%)	12 (22.6%)	15 (34.1%)	13 (26.5%)	
**Lung**						0.390
**no**	36 (85.7%)	41 (93.2%)	43 (81.1%)	38 (86.4%)	39 (79.6%)	
**yes**	6 (14.3%)	3 (6.82%)	10 (18.9%)	6 (13.6%)	10 (20.4%)	
**Heart**						0.063
**no**	23 (54.8%)	27 (61.4%)	35 (66.0%)	33 (75.0%)	23 (46.9%)	
**yes**	19 (45.2%)	17 (38.6%)	18 (34.0%)	11 (25.0%)	26 (53.1%)	
**BMI**	26.2 (5.80)	26.1 (5.81)	27.8 (6.21)	26.5 (6.10)	24.5 (5.22)	0.171
**Smoking**						0.002
**No**	34 (81.0%)	22 (50.0%)	41 (77.4%)	25 (56.8%)	38 (77.6%)	
**Yes**	5 (11.9%)	9 (20.5%)	8 (15.1%)	14 (31.8%)	7 (14.3%)	
Missing	3 (7.14%)	13 (29.5%)	4 (7.55%)	5 (11.4%)	4 (8.16%)	

Table S8

Linear regression model for Wound infections over the years. Switzerland, 2017 - 2021			
**Predictors**	**Odds Ratios**	**CI**	**p-value**
(Intercept)	-0.03	-0.05 – -0.02	<0.001
2018	0.01	-0.00 – 0.02	0.106
2019	0.02	0.01 – 0.04	<0.001
2020	0.02	0.01 – 0.03	0.004
2021	0.02	0.01 – 0.03	0.002
Age	0.00	-0.00 – 0.00	0.482
Sex (female)	-0.00	-0.01 – 0.01	0.905
PCCL	0.02	0.01 – 0.02	<0.001
Observations	7309		
R2/ R2 adjusted	0.033 / 0.032		

Table S9

Linear regression model for pneumonia over the years. Switzerland, 2017 - 2021			
**Predictors**	**Odds Ratios**	**CI**	**p-value**
(Intercept)	-0.04	-0.05 – -0.02	<0.001
2018	0.00	-0.01 – 0.01	0.702
2019	0.01	-0.00 – 0.02	0.093
2020	0.00	-0.01 – 0.01	0.451
2021	-0.00	-0.01 – 0.01	0.363
Age	0.00	0.00 – 0.00	<0.001
Sex (female)	-0.01	-0.01 – -0.00	0.010
PCCL	0.01	0.01 – 0.02	<0.001
Observations	7309		
R2/ R2 adjusted	0.038 / 0.038		

Figure S1

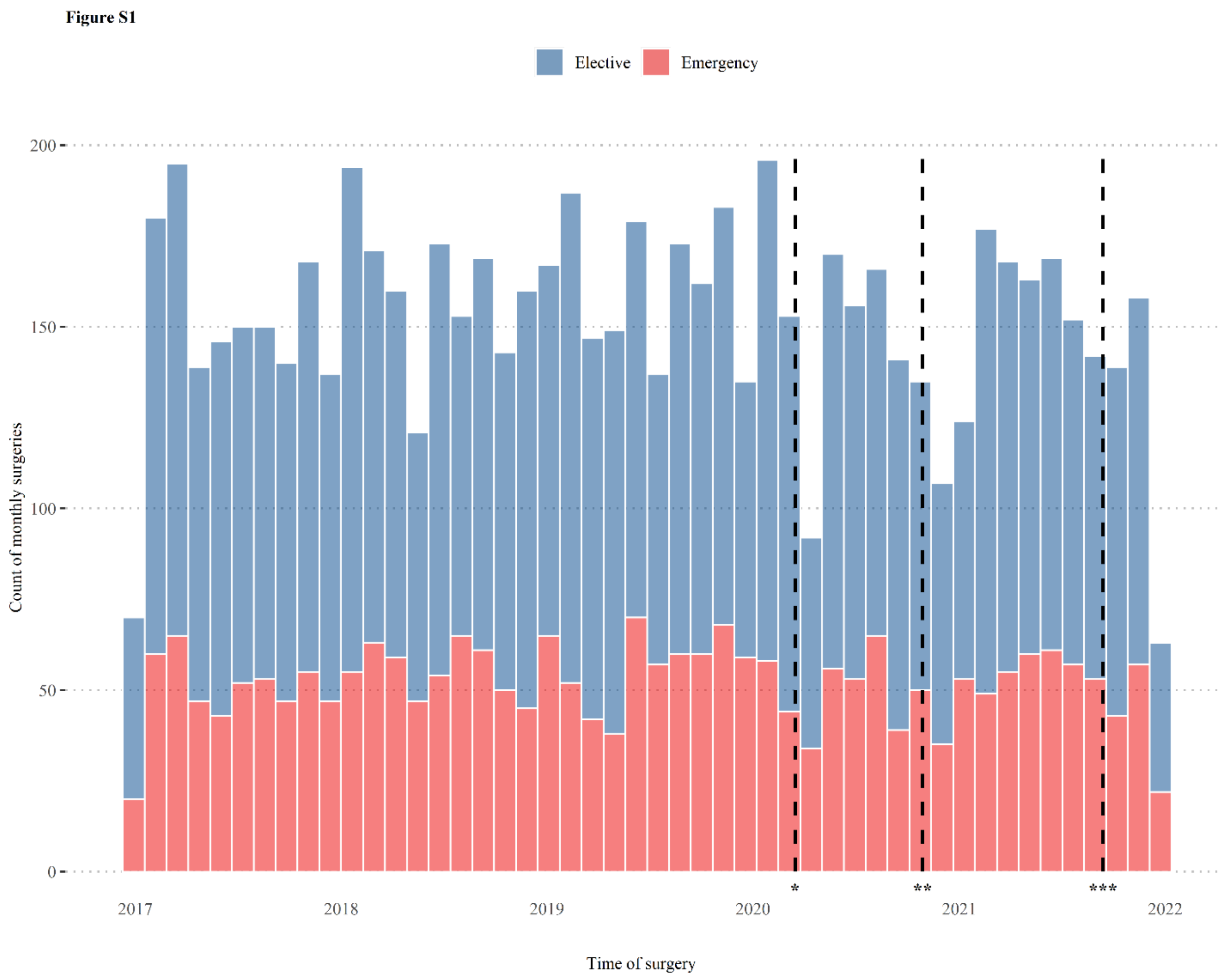

Figure S1 – Number of surgeries over the years. Switzerland, 2017 - 2021
All 7309 performed procedures between January 1, 2017, and December 31, 2021, subdivided into elective or emergency indication. * March 16, 2020, first lockdown, ** October 19, 2020, mandatory mask wearing, *** September 19, 2021, implementation of national certificate

Figure S2

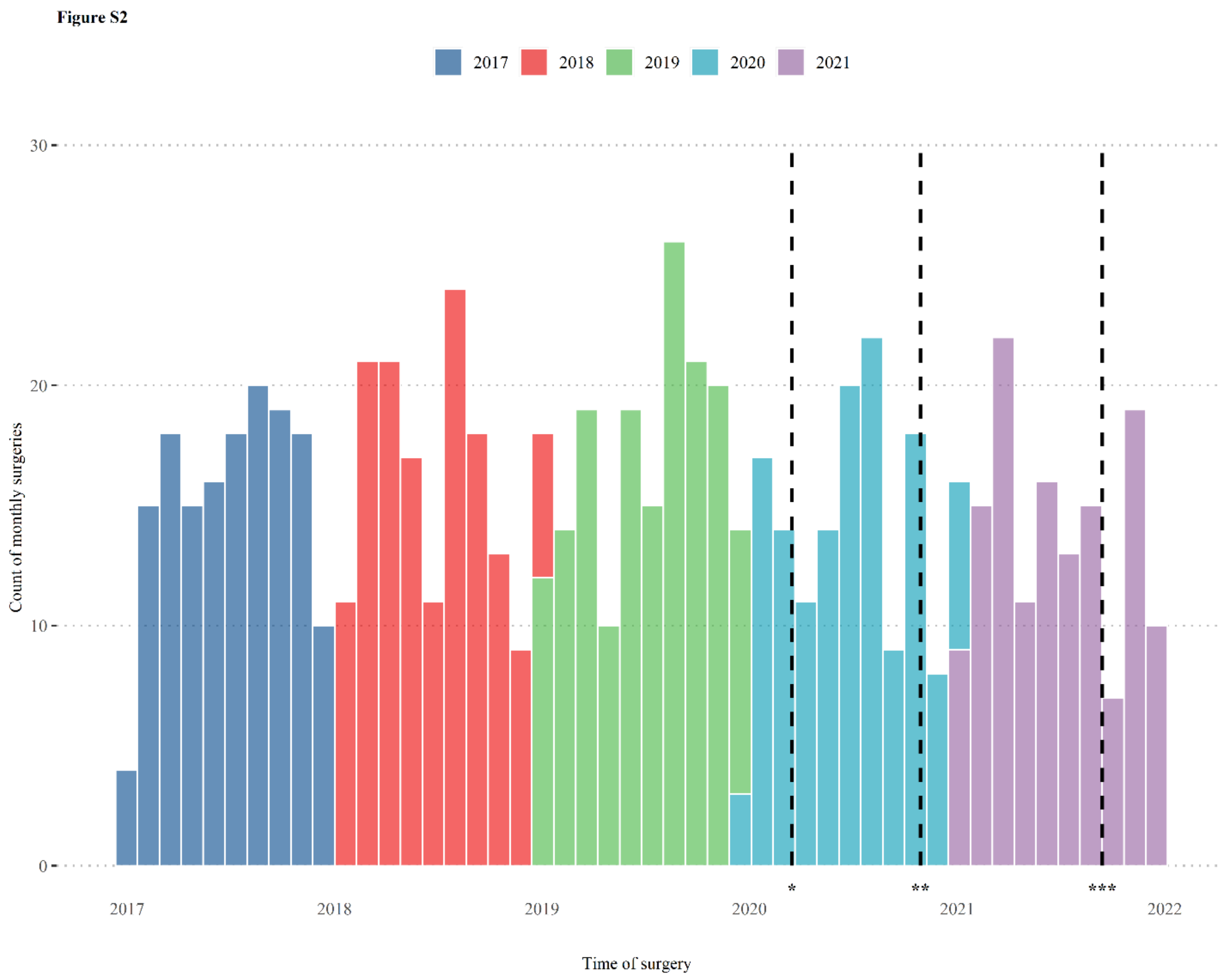

Figure S2 – Emergency appendectomies over the years. Switzerland, 2017 - 2021
All 751 emergency appendectomies performed between January 1, 2017, and December 31, 2021. * March 16, 2020, first lockdown, ** October 19, 2020, mandatory mask wearing, *** September 19, 2021, implementation of national certificate

Figure S3

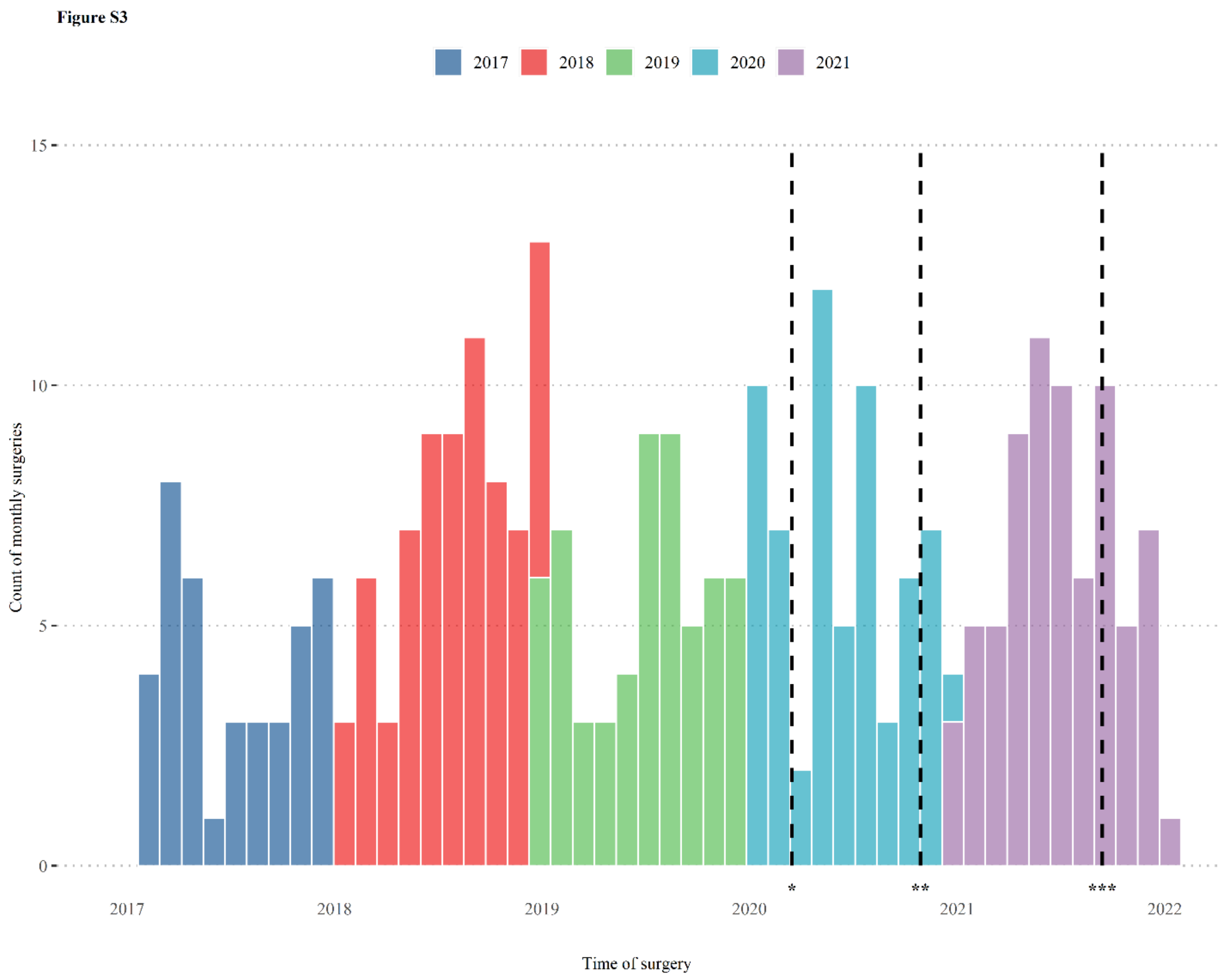

Figure S3 – Emergency cholecystectomies over the years. Switzerland, 2017 - 2021
All 302 emergency cholecystectomies performed between January 1, 2017, and December 31, 2021. * March 16, 2020, first lockdown, ** October 19, 2020, mandatory mask wearing, *** September 19, 2021, implementation of national certificate

Figure S4

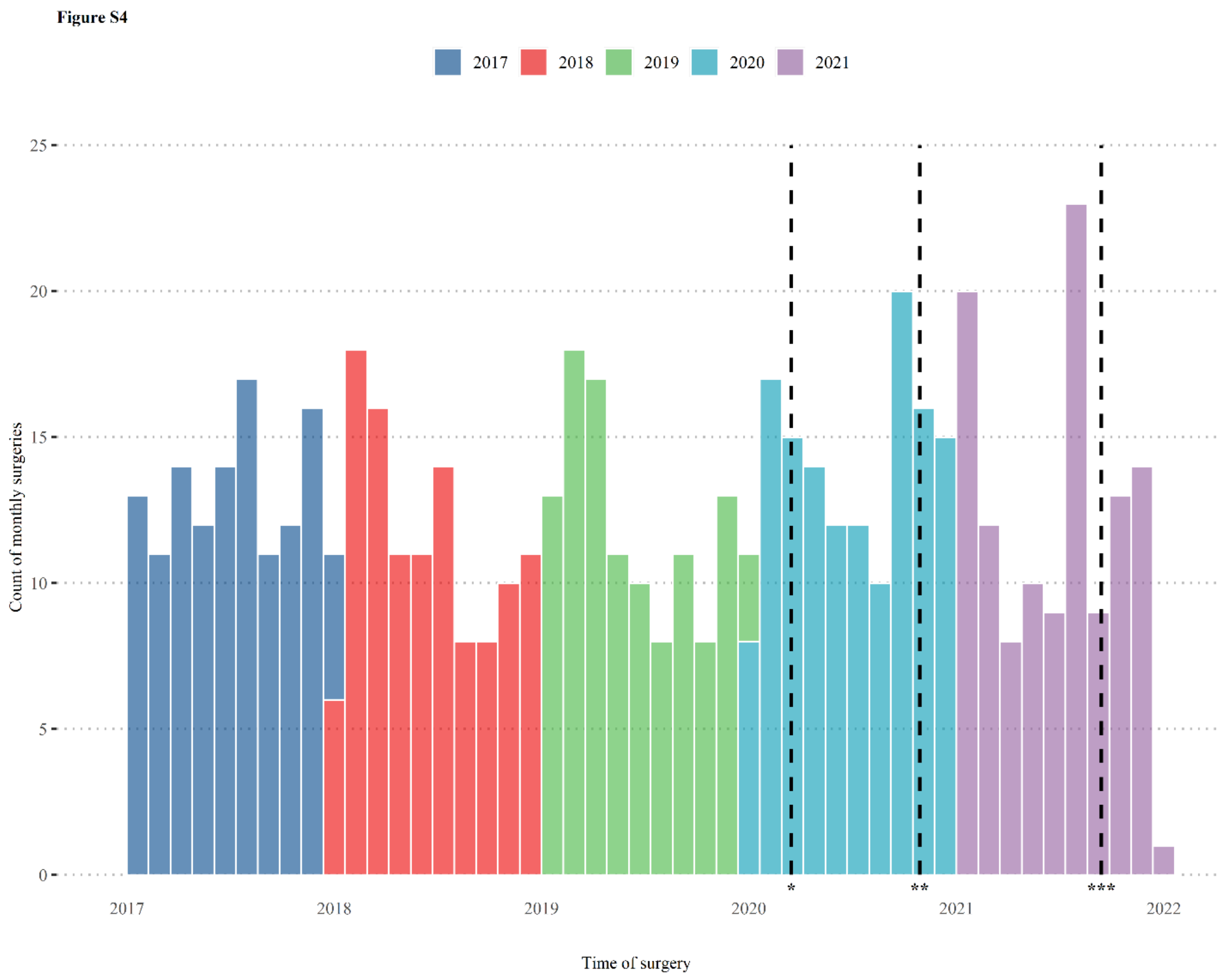

Figure S4 – Elective liver and pancreatic procedures over the years. Switzerland, 2017 - 2021
All 608 elective liver and pancreatic procedures performed between January 1, 2017, and December 31, 2021. * March 16, 2020, first lockdown, ** October 19, 2020, mandatory mask wearing, *** September 19, 2021, implementation of national certificate

Figure S5

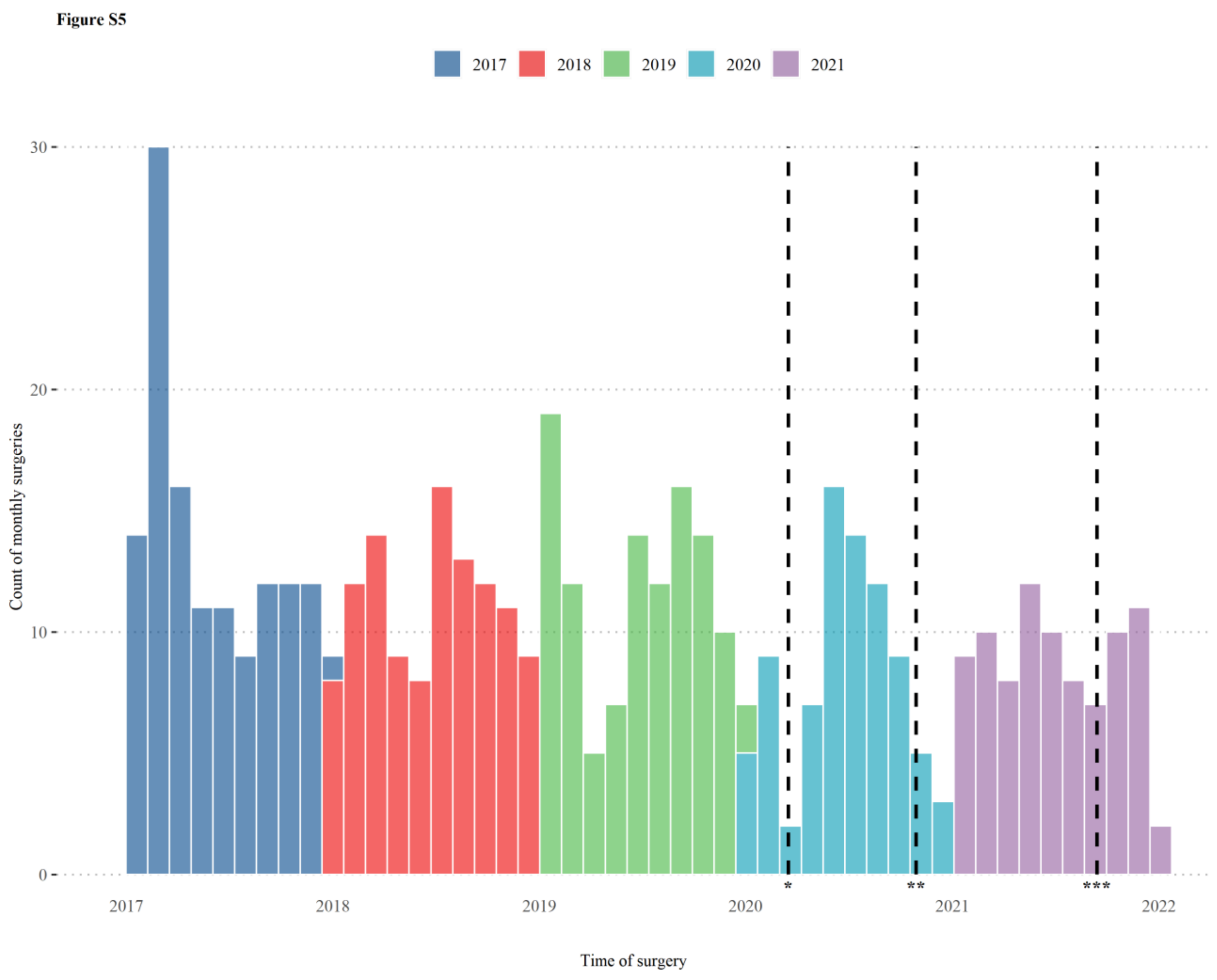

Figure S5 – Gastric bypasses over the years. Switzerland, 2017 - 2021
All 520 elective gastric bypasses performed between January 1, 2017, and December 31, 2021. * March 16, 2020, first lockdown, ** October 19, 2020, mandatory mask wearing, *** September 19, 2021, implementation of national certificate
